# Exploring the Mechanism of Action and Potential Targets of Saorilao‐4 Decoction in the Treatment of Pulmonary Fibrosis in Rats by Metabolomics

**DOI:** 10.1002/fsn3.4633

**Published:** 2025-01-31

**Authors:** Jiali Liu, Xinni Song, Xinyue Fu, Shufang Niu, Hong Chang, Songli Shi, Meiqing Yang, Peng Wang, Wanfu Bai

**Affiliations:** ^1^ Department of Pharmacy Baotou Medical College Baotou China; ^2^ The Second Affiliated Hospital of Baotou Medical College Baotou China; ^3^ Institute of Bioactive Substance and Function of Mongolian Medicine and Chinese Materia Medica Baotou Medical College Baotou China

**Keywords:** fatty acid metabolism, metabolomics, pulmonary fibrosis, Saorilao‐4 decoction Monk's medicine, sphingolipid metabolism

## Abstract

Pulmonary fibrosis (PF) is a chronic progressive disease marked by alveolar epithelial cell damage. Saorilao‐4 decoction (SRL), a traditional Mongolian prescription, has demonstrated therapeutic effects on PF, though its mechanism of action remains elusive. This study used a bleomycin‐induced fibrosis rat model to evaluate SRL's effects by measuring inflammatory factors, assessing fibrosis‐related indices, and performing histopathological lung examinations. Serum metabolite levels in the experimental groups were measured using high‐performance liquid chromatography coupled with mass spectrometry. Data analysis involved principal component and partial least‐squares discriminant analyses, followed by functional enrichment analysis of differential metabolites. SRL significantly ameliorated alveolar interstitial injury, fibrosis, and metabolic disorders induced by bleomycin. Additionally, we identified 71 metabolic components related to PF progression, including sphingolipids and fatty acids. Administration of SRL affected 59 metabolic components involved in purine, cysteine and methionine, and arginine and proline metabolisms. Specifically, SRL regulated the levels of hexadecanoic acid, S‐adenosylmethionine, 3‐oxopalmitoyl coenzyme A, and dodecanoic acid metabolites, thereby improving the metabolic course of PF. In conclusion, this study offers insights into the potential mechanisms of SRL in treating PF from a metabolomics perspective. It provides valuable information for its clinical application.

## Graphical abstract

Saorilao‐4 decoction (SRL), a Mongolian prescription, has shown therapeutic effects on pulmonary fibrosis (PF) in a rat model. Seventy‐one metabolic components, including sphingolipids and fatty acids, were identified in relation to PF progression. SRL influenced 59 metabolic components, particularly improving the metabolism of hexadecanoic acid and other key metabolites, suggesting a potential mechanism for its therapeutic action.

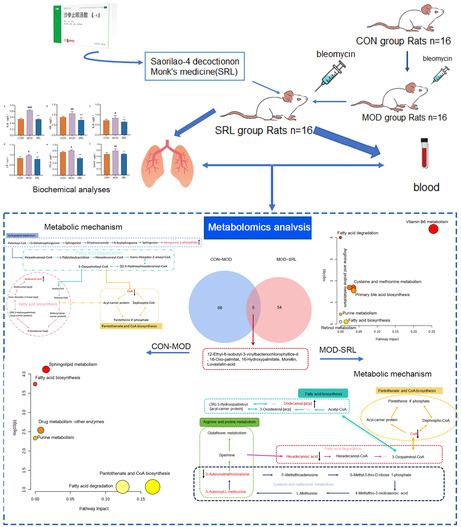

## 1 Introduction

Pulmonary fibrosis (PF) is a diffuse parenchymal lung abnormality characterized by dyspnea, cough, hypoxemia, and impaired gas exchange, eventually leading to respiratory failure. PF develops from repeated localized injuries to the alveolar epithelium, leading to abnormal fibroblast growth, accumulation of extracellular matrix, and remodeling of the interstitium. Autophagy, apoptosis, senescence, and inflammation all contribute to the development of PF (Li et al. 2022a). The increasing global incidence of PF has resulted in increased hospitalization and mortality rates, severely impacting patients' quality of life (Ryerson and Kolb 2018). Current clinical treatments, such as pirfenidone and nintedanib, inhibit fibroblast proliferation induced by tumor growth factor beta 1 (TGFβ1) (Roach et al. 2021), aiming to reduce spirometric decline and the risk of acute exacerbations in PF patients (Amati et al. 2023).

However, these medications often result in numerous early adverse reactions, including cardiovascular complications and respiratory impairment (Fournier et al. 2022). Long‐term adherence to these treatments is notably poor in subnational populations (Song et al. 2020), particularly affecting effectiveness in Asian populations (Kato et al. 2021; Pan et al. 2023; Meyer and Decker 2017). Recent clinical trials have shown that traditional Chinese medicine (TCM) provides unique therapeutic approaches to PF. TCM is noted for its high efficiency, low toxicity, minimal side effects, and cost‐effectiveness, attracting significant attention from both medical professionals and patients (Liu et al. 2022). TCM is advantageous due to its gentle nature, reduced gastrointestinal irritation, simple preparation, rapid absorption, and quick onset of action (Zhi et al. 2020).

The Mongolian proprietary medicine Saorilao‐4 decoction (SRL), also referred to as “Bei Sha Shen eSi Wei Tang,”, “Sha Shen Cough Soup San,” or “Saorilao Xi Tang,” originates from the Sea of Medical Laws and was included in the Standard of Mongolian Medicines of Inner Mongolia in 1984 (Heng, Lian, and Mu 2022). This formula, which includes *Glehnia littoralis* Fr. Schmidt ex Miq., *Polygonum bistorta* L., *Glycyrrhiza uralensis* Fisch., and Laccifer lacca Kerr (Lacciferidae Cockerell), is commonly used in Mongolian medicine to treat coughs, yellow or bloody sputum due to lung heat, lung pain, colds, and chest and back tingling caused by blood heat, demonstrating remarkable curative effects (Qing, Kong, and Xin 2023). SRL is typically administered for 2 weeks per treatment course (Ma 2019), yielding positive outcomes in lung disease after 2–3 treatment courses (Tu and Na Ren 2018).

Previous research using the tracheal phenol red secretion method in mice demonstrated that SRL has anti‐inflammatory and antitussive effects (Lan and Jin 2016). Additionally, SRL has been shown to significantly ameliorate pathological changes in rats with PF, delaying and reversing fibrosis progression through mechanisms such as inflammatory response inhibition, lipid peroxidation mitigation, downregulation of growth factor beta 1 and suppressor of mothers against decapentaplegic (Smad) 3 mRNA, and upregulation of Smad7 mRNA expression (Bai, Liu, and Li 2021). Despite these findings, the metabolomic mechanism of SRL in treating PF remains unexplored.

Systems biology techniques like metabolomics, genomics, and proteomics are extensively used to study the comprehensive functions and interconnections of organisms, identify biomarkers, and reveal changes across various biological stages. This approach offers a new way to explore the biological foundations of diseases (Santolini and Barabási 2018). Metabolomics research focuses on disease‐specific samples and has gained widespread attention for providing new insights into the fundamental aspects of pathomechanisms and pharmacodynamics (du Preez, Luies, and Loots 2019). This study aims to investigate the mechanism of SRL in PF using metabolomic and bioinformatics analyses. Serum samples from rats with PF treated with SRL were collected to examine differential serum metabolites, analyze the decoction's effects on associated metabolic pathways, and identify potential markers and pathways linked to its efficacy in PF. The findings provide valuable data for treating PF with SRL and offer new perspectives for developing ethnomedicine in managing this condition.

## 2 Materials and Methods

### 2.1 Main Drugs and Reagents

SRL (batch no. 20200414, 3 g per bag, mass ratio of *Glehnia littoralis* Fr. Schmidt ex Miq.: *Polygonum bistorta* L.: *Glycyrrhiza uralensis* Fisch: Laccifer lacca Kerr (Lacciferidae Cockerell) 5:3:3:3) was procured from the National Center of Mongolian Pharmaceutical Preparation of the Inner Mongolia International Mongolian Medical Hospital. We obtained bleomycin for injection (batch no. SL30191404, 100 mg/mL) from Beijing Koolaibao Science and Technology Co. Ltd. The hematoxylin–eosin (HE) staining kit (batch no. 20191231) was sourced from Beijing Biolab Technology Co. Ltd. We acquired the following detection kits from Nanjing Jianjian Bioengineering Research Institute: Masson's Trichrome stain (batch no. 20191025), interleukin (IL)‐1β (batch no. CK‐E30206), IL‐6 (batch no. CK‐E30219), hyaluronidase (HA; batch no. CK‐E30811), laminin (LN; batch no. CK‐E30254), procollagen III (PC‐III; batch no. CK‐E31310), and collagen IV (Col‐IV; batch no. CK‐E34380). We purchased sodium chloride injection (batch no. 2012056G, 1.8 g per 100 mL) from Shandong Hualu Pharmaceutical Company Limited. Methanol, used for mass spectrometry, was supplied by Thermo Fisher Scientific (USA). Distilled water was utilized throughout the experiments.

### 2.2 Animal and Sample Collection

Forty‐eight healthy adult male specific pathogen‐free grade Sprague–Dawley rats (180–250 g) were procured from the China Academy of Food and Drug Control (animal production license no. SCXK 2017‐0005). The study received ethical approval from the Committee on Ethics in Animal Experiments of Baotou Medical College (no. 2022‐96). The rats were housed in a controlled environment with a standard 12:12 h light: dark cycle, constant humidity (50 ± 5)%, and temperature (22°C–25°C), with unrestricted access to food and water. A 7‐day acclimatization period preceded the experiment.

**FIGURE 1:**
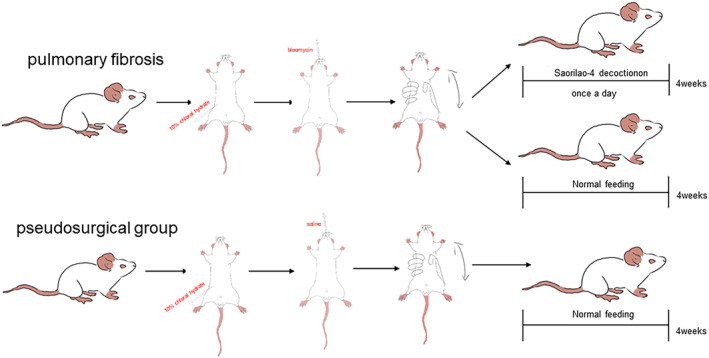
Preparation of experimental models and drug treatment processes.

Rats were divided into three groups: the control group (CON) (*n* = 16), the PF modeling group (MOD) (*n* = 16), and the SRL‐treated group (SRL) (*n* = 16). The methods of inducing PF are referred to in the literature (Shen et al. 2021; Chen et al. 2016; Li, Jin, and Ji 2023). The modeling of idiopathic PF in rats was considered successful when the extent of lung injury exceeded 1/2 of the total lung region (Ashcroft, Simpson, and Timbrell 1988). After the acclimatization period, rats in the experimental groups were anesthetized by the intraperitoneal administration of 10% pentobarbital sodium (3.8 mL × kg^−1^) and placed supine on the table, with fixed head and limbs. Bleomycin (5 mg/kg, approximately 0.2 mL) was slowly injected into the gap of the cartilage ring of the trachea through a blunt‐tipped lumbar puncture needle in both the MOD and SRL groups. Subsequently, 0.2 mL of air was introduced into the windpipe two to three times, followed by 0.2 mL of air injection into the trachea two to three times. The rats were quickly placed upright and rotated to ensure even lung medication distribution (Zeng et al. 2023). The CON group received an equal volume of saline in the trachea under the same conditions. After natural awakening, the animals were returned to their cages for routine care (Figure 1).

Post‐modeling, rats in the CON and MOD groups received saline (1 mL/100 g) via gavage, while rats in the SRL group were administered the corresponding dose of the SRL in saline solution once daily (1 mL/100 g) (Bai, Liu, and Li 2021). Rat health and mortality were monitored throughout the study. After 4 weeks of treatment, at 24 h following the final administration, the rats were anesthetized with an intraperitoneal injection of sodium pentobarbital solution (3%, 30 mg/kg). Once confirmed to be unresponsive to pain stimuli, the rats were placed ventral‐side up on a dissecting board, with limbs and upper incisors secured. Sterilization from the neck to the abdomen was performed using 75% alcohol. Longitudinal incisions were made with tissue scissors, opening the chest cavity by cutting upward from the median abdominal line. Immediate observations were conducted on the lungs' color, texture, morphology, and volume. Subsequently, 5 mL of blood was drawn from the abdominal aorta (Lv et al. 2023), and the serum was stored at −80°C. Tissue from the left‐lower lobe of the lungs was fixed in a 4% paraformaldehyde solution, while another portion from the right lungs was rapidly frozen using liquid nitrogen and stored at −80°C.

### 2.3 Histomorphological Observation of Rat Lung

The upper‐right lung lobe was fixed in a 4% paraformaldehyde solution and embedded in paraffin. Thin sections (5 μm) were prepared using a paraffin sectioning apparatus (Finesse E+, Thermo Fisher Scientific) and stained with HE and Masson's Trichrome staining to observe pathological changes under a microscope (BX43; Olympus).

### 2.4 Biochemical Marker Assessment

The serum, thawed from −80°C in a refrigerator at 4°C, was processed according to the manufacturer's instructions. Levels of IL‐1β, IL‐6, HA, LN, PC‐III, and Col‐IV were measured using an Olympus AU640 autoanalyzer (Olympus, Japan).

### 2.5 Serum Metabolomics Studies

#### 2.5.1 Sample Preparation

A total of 100 μL of the liquid sample was carefully pipetted into a 1.5‐mL centrifuge tube, followed by the addition of 400 μL of the extraction solution (acetonitrile: methanol = 1:1). The mixture was vortexed for 30 s to ensure thorough homogenization. Ultrasonication was performed at 40 kHz and 5°C for 30 min to enhance the extraction process. After ultrasonication, the sample was allowed to settle for 30 min at −20°C and then centrifuged for 15 min at 13,000 *g* and 4°C. The supernatant was transferred to a new container, subjected to nitrogen drying to remove residual solvents, and stored at −80°C for liquid chromatography–tandem mass spectrometry (LC–MS/MS) analysis. QC samples, consisting of 20 μL of supernatant, were injected into the LC–MS/MS system (UPLC‐Q‐Exactive Quadrupole‐Electrostatic Field Orbitrap High‐Resolution Mass Spectrometer, Thermo Fisher [Shanghai] Instruments Co.) at regular intervals (every 9–10 samples) to monitor and ensure the reliability of the analytical process.

#### 2.5.2 Ultra‐Performance Liquid Chromatography–Tandem Quadrupole Time‐of‐Flight Mass Spectrometry (UPLC‐Q‐TOF/MS) Analysis

UPLC–MS was conducted using an ACQUITY UPLC HSS T3 C18 column (2.1 × 100 mm, 1.8 μm, Waters) with a mobile phase comprising two solvents: (A: 0.1% formic acid plus formic acid in water (0.1%); B: 0.1% formic acid plus acetonitrile: isopropanol [1:1, v/v]). The solvent gradient conditions during chromatographic analysis were (a) 0–3 min, 95% (A): 5% (B) to 80% (A): 20% (B); (b) 3–9 min, 80% (A): 20% (B) to 5% (A): 95% (B); (c) 9–13 min, 5% (A): 95% (B) to 5% (A): 95% (B); (d) 13–13.1 min, 5% (A): 95% (B) to 95% (A): 5% (B); (e) 13.1–16 min, 95% (A): 5% (B) to 95% (A): 5% (B) for system equilibration. The injection volume for each sample was 20 μL, with a flow rate of 0.4 mL/min, and the column temperature was maintained at 40°C. Samples were stored at −4°C during the analysis.

### 2.6 Bioinformatics Analysis

The MS software was used to perform peak extraction and peak alignment of the ion peaks in different samples to obtain the raw abundance information of each metabolite ion in the samples. For the ions detected by XCMS, the open source software metaX was firstly used to match the first level m/z of the substances with the databases such as HMDB and KEGG to get the first‐level identification results and to obtain the metabolite identification results with higher credibility.

Hematology metabolic spectra of rats (*n* = 6 per group) were analyzed using MarkerLynx software (v. 4.1). Raw data underwent preprocessing steps, including filtering, complementary value assignment, normalization, logarithmic transformation, and data reduction. Serum metabolic data from the CON, MOD, and SRL groups were processed using ProgenesisQI software to obtain mass spectrometry matrix information, which was subsequently analyzed using SPSS 26.0. Measurement data are presented as mean ± standard deviation ($\bar{x}$ ± s). Multiple‐group comparisons were conducted using one‐way ANOVA, while within‐group pairwise comparisons utilized the rank‐sum test.

Multivariate analysis involved principal component analysis (PCA) with partial least‐squares discriminant analysis (PLS‐DA) visualization of clustering and grouping, performed on data exported from QI software using EZinfo 3.0 for Waters software to initially assess model validity. Permutation testing (PT) further validated the model to distinguish between groups (Tan et al. 2022). Differential metabolites were identified based on a variable importance in projection (VIP) score > 1 and a univariate statistical analysis *p* < 0.05 (Li et al. 2022b). Volcano plots visually represented differential metabolites between groups. For these identified differential metabolites, receiver operating characteristic (ROC) curves were generated, and the area under the curve (AUC) was calculated to evaluate their validity. Histograms depicted the classification results of differential metabolic component analysis. Common differential metabolic components between the CON and MOD groups and the MOD and SRL groups were analyzed to elucidate their modulation in PF.

A heat map of differential abundance visualized the distribution of differential metabolites in each group. Additionally, correlation heat maps analyzed the role of SRL in treating idiopathic PF. Kyoto Encyclopedia of Genes and Genomes (KEGG) differential enrichment analysis of metabolites determined the pathways impacted by SRL in PF, providing a comprehensive understanding of metabolite level changes associated with PF.

## 3 Results

### 3.1 Histological Changes in Rat Lung

**FIGURE 2:**
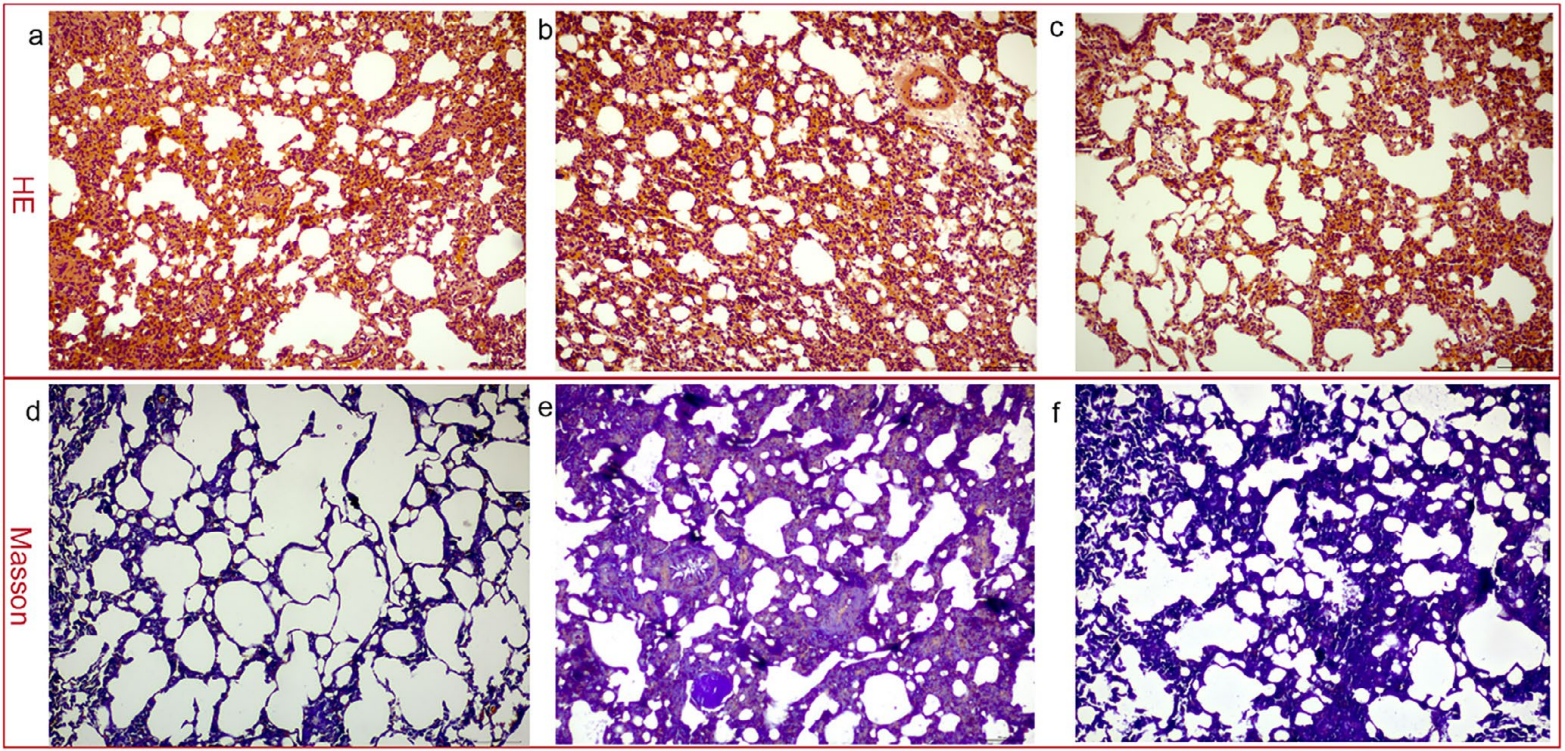
Micrographs of HE‐ and Masson's‐stained lung tissues. (a, d), control group, (b, e), model group, and (c, f), Saorilao‐4 decoction‐treated group.

Micrographs of HE‐ and Masson's‐stained lung tissues are depicted in Figure 1. HE staining of lung tissues from the CON group revealed a well‐structured pattern, free of fatty degeneration and with minimal inflammatory response (Figure 2a). In stark contrast, the MOD group exhibited a significant inflammatory response. This was characterized by thickened alveolar septa and substantial infiltration of inflammatory cells in the interstitial spaces of the alveoli and around the bronchial tubes (Figure 2b). The SRL group demonstrated significant improvement, with reduced interstitial infiltration of inflammatory cells and enhanced alveolar structure compared to the MOD group (Figure 2c).

Masson's staining showed that lung tissues in the CON group had a normal structure, with no visible blue staining (Figure 2d). The MOD group exhibited extensive blue staining, indicating considerable fibrosis (Figure 2e). In contrast, the SRL group showed a noteworthy reduction in blue‐stained areas, suggesting an improvement in fibrosis (Figure 2f).

### 3.2 Measurement of Inflammatory Factors and Lung Function Biomarker Levels in Lung Function

**FIGURE 3:**
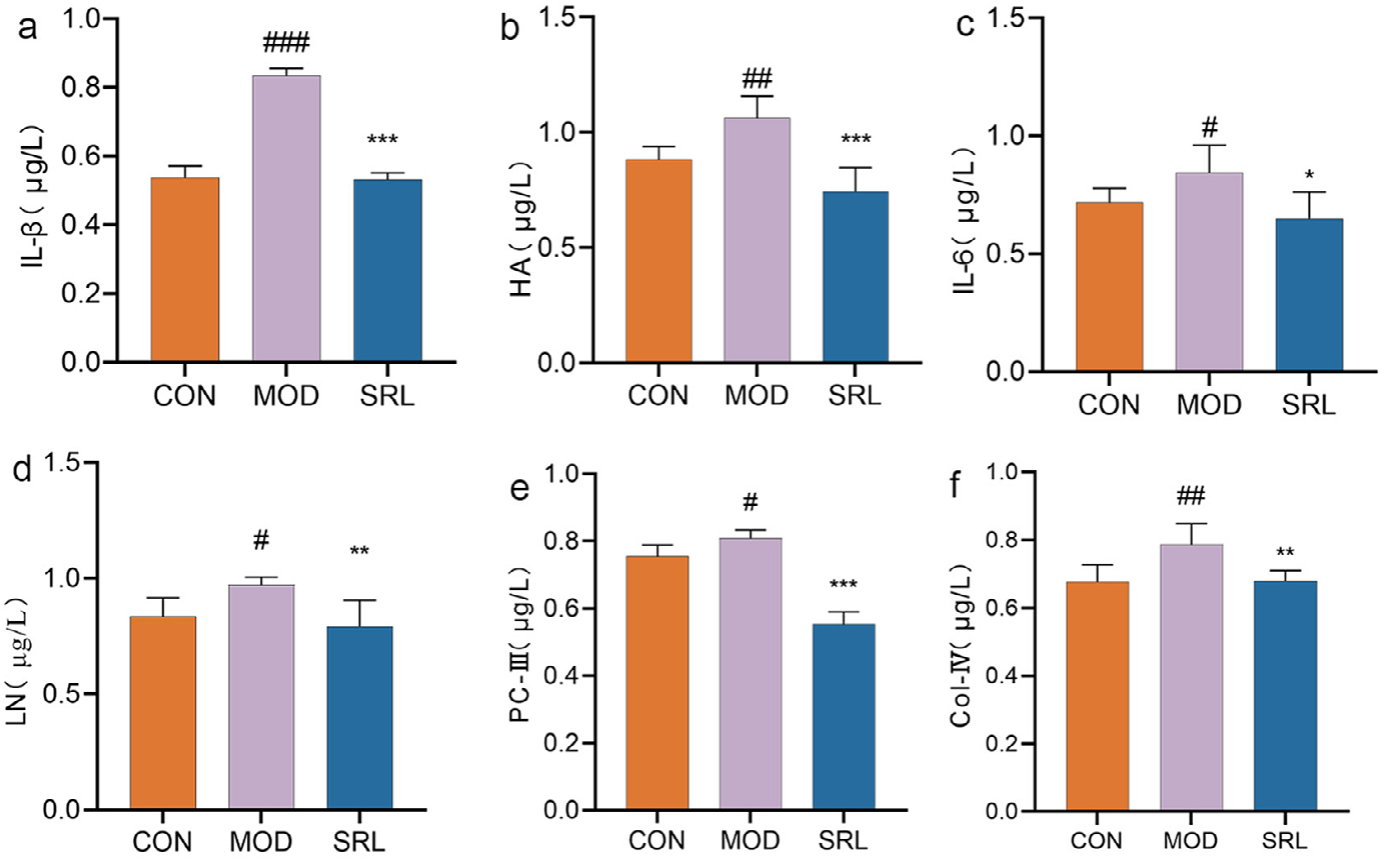
Effects of Saorilao‐4 decoction on the contents of inflammatory factors and fibrosis‐related indices in the lung tissue of specific pulmonary fibrosis model rats. Comparison between model group and normal control group, ###*p* < 0.001; ##*p* < 0.01; #*p* < 0.05; SRL group was compared with the model group, ***p < 0.001; * * *p* < 0.01; * *p* < 0.05. CON: control group; MOD: model group; SRL: Shao Riliao 4 decoction treatment group.

The levels of IL‐1β, IL‐6, HA, LN, PC‐III, and Col‐IV in the lungs were significantly higher in the MOD group than in the CON group (*p* < 0.01) (Figure 3). The SRL group showed significantly lower levels of IL‐1β, IL‐6, HA, LN, PC‐III, and Col‐IV compared to the MOD group (*p* < 0.01).

### 3.3 Effect of Saorilao‐4 Decoction‐Treated Group on Serum Metabolic Profiles of Experimental Rats

**FIGURE 4:**
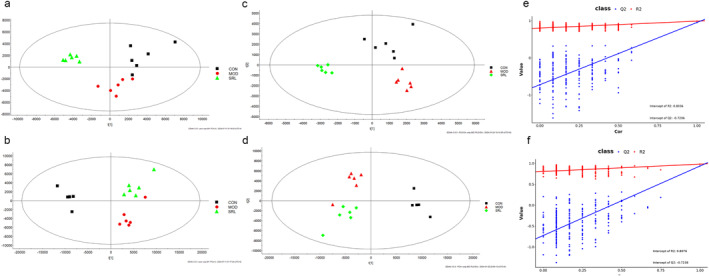
Plots of PCA and PLS‐DA scores of metabolites in serum samples. (a, b) PCA scores plotted in negative and positive modes for the CON and MOD groups. (c, d) PLS‐DA scores plotted in negative and positive modes. (e, f) Validation of the PLS‐DA model through a class alignment test in positive and negative modes. CON, control group; MOD, model group; SRL, Saorilao‐4 decoction‐treated group; PCA, principal component analysis; PLS‐DA, partial least‐squares discriminant analysis.

We examined the metabolic profiles of rat serum samples from different groups using the UPLC–QTOF–MS method, employing both positive and negative electrospray ionization (ESI) modes. We analyzed data from the CON, MOD, and SRL groups using unsupervised PCA. The PCA results (Figure 4a,b) revealed distinct separations among the CON, MOD, and SRL groups into three independent regions, indicating excellent group differentiation. Furthermore, the strong clustering within each group indicates stability and reproducibility in the instrumentation and data acquisition, which enhances the reliability of the experimental data. To further evaluate metabolic profile variability, PLS‐DA was applied (Figure 4c,d) and validated with a permutation test (Figure 4e,f). In the positive mode, the R2 and Q2 values were 0.81 and −0.72, respectively, while in the negative mode, they were 0.80 and −0.72, respectively. Q2 estimates the model's predictive power, calculated through cross‐validation (CV), while *R* ^2^ indicates the model's fit. The values of *R* ^2^ and Q2 are close to 1, indicating that the developed model shows good adaptive and predictive performance.

### 3.4 Screening and Characterization of Differential Metabolites

**FIGURE 5:**
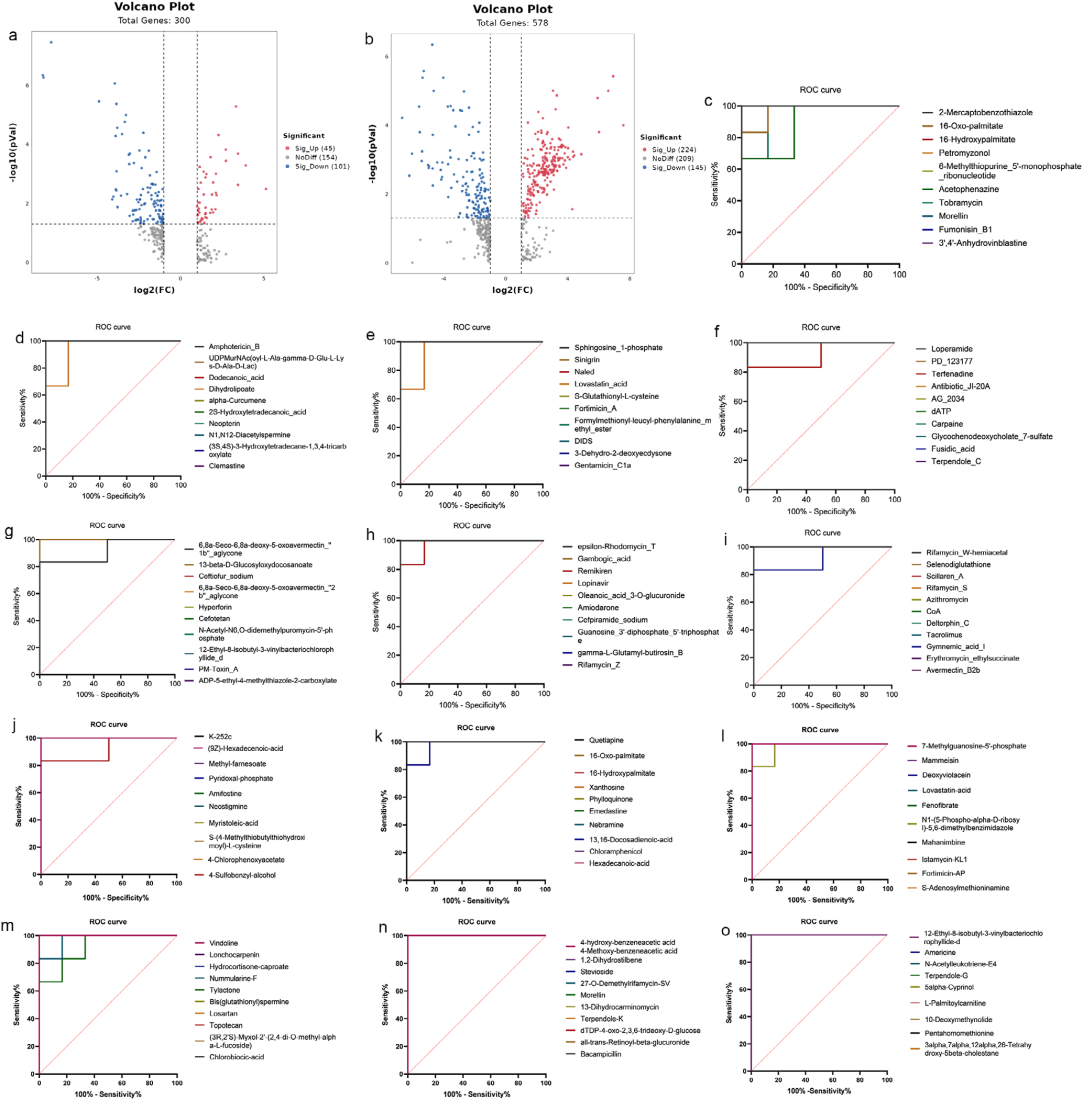
Score plots and ROC curves of serum sample metabolites. (a) Volcano plot showing CON versus MOD in a negative mode for differential metabolites. Volcano plots are a commonly used graph to demonstrate differences in gene or metabolite expression. (b) Similar to panel (a), showing the volcano map in a positive mode. Panels (c–i) demonstrate the ROC curve analysis of 71 potential differential biomarkers between CON and MOD. (j–o) ROC analysis of the 59 potential differential biomarkers between the MOD and SRL groups. CON, control group; MOD, model group; SRL, SRL‐treated group; ROC, receiver operating characteristic curve, area under the curve (AUC) is a numerical metric used to quantify the overall performance of a model; the higher the AUC value, the better the model's predictive ability.

We analyzed the resulting data, which included 878 serum constituents, for statistical significance and the magnitude of change, as shown in volcano plots (Figure 5a,b). In the negative ion mode, 45 constituents were upregulated and 101 were downregulated. In the positive ion mode, 224 constituents were upregulated and 145 were downregulated. The levels of the remaining 363 components showed no significant changes.

To clarify the differences between the models and confirm the variations in serum component levels, we identified differential metabolites in both the CON and MOD groups and the MOD and SRL groups. Combined analysis revealed 71 differential serum metabolites between the CON and MOD groups and 59 between the MOD and SRL groups. We performed ROC analyses to experimentally verify the diagnostic potential of the identified differential metabolites (Figure 5c–o). The AUC ranges from 0 to 1. Larger AUC values indicate better classification performance of the model. The results indicated that the AUC values for all 130 potential biomarkers exceeded 0.8, suggesting favorable diagnostic accuracy.

Of the 71 differential metabolites identified between the CON and MOD groups, 46 were detected in positive ionization mode and 25 in negative ionization mode (Table 1).

**TABLE 1:** The 71 differential metabolites between the CON and MOD groups.

No	Metabolites	Source	m/z	Retention time[s]	MS1keggID	Ion mode	VIPa	Regulated (M/C)	Regulated (S/M)
1	2‐Mercaptobenzothiazole	Endogenous	165.9796	3.540966667	C14437	ESI—	2.570221	↓	
2	16‐Oxo‐palmitate	Exogenous	269.2126	4.389716667	C19614	ESI—	1.083693	↑	↓
3	16‐Hydroxypalmitate	Endogenous	271.2282	5.080716667	C18218	ESI—	1.14643	↑	↓
4	Petromyzonol	Endogenous	393.3004	6.23915	C16258	ESI—	1.456223	↓	
5	6‐Methylthiopurine‐5′‐monophosphate‐ribonucleotide	Exogenous	394.0556	0.866233333	C16615	ESI—	1.462777	↓	
6	Acetophenazine	Exogenous	427.2191	2.70115	C06807	ESI—	1.744187	↑	
7	Tobramycin	Exogenous	483.2812	3.14245	C00397	ESI—	1.799464	↑	
8	Morellin	Exogenous	560.2638	4.00355	C10085	ESI—	2.534897	↑	↓
9	Fumonisin‐B1	Endogenous	720.3773	2.8016	C19241	ESI—	2.641306	↓	
10	3′,4′‐Anhydrovinblastine	Exogenous	808.4304	2.840616667	C11641	ESI—	2.546393	↓	
11	Amphotericin‐B	Exogenous	939.5058	2.882783333	C06573	ESI—	2.620882	↓	
12	UDPMurNAc(oyl‐L‐Ala‐gamma‐D‐Glu‐L‐Lys‐D‐Ala‐D‐Lac)	Endogenous	1189.296	4.624466667	C20869	ESI+	1.331193	↓	
13	Dodecanoic acid	Endogenous	218.2114	4.574966667	C02679	ESI—	1.683597	↑	
14	Dihydrolipoate	Endogenous	231.0469	0.947125	C02147	ESI—	1.417235	↓	
15	alpha‐Curcumene	Exogenous	241.1366	4.317558333	C09649	ESI+	1.637846	↓	
16	2S‐Hydroxytetradecanoic‐acid	Exogenous	262.2379	4.610358333	C13790	ESI—	1.395119	↑	
17	Neopterin	Exogenous	271.1148	4.279458333	C05926	ESI+	1.604069	↑	
18	N1,N12‐Diacetylspermine	Endogenous	309.2271	4.993291667	C03413	ESI+	1.641316	↓	
19	(3S,4S)‐3‐Hydroxytetradecane‐1,3,4‐tricarboxylate	Exogenous	347.2082	4.29785	C04529	ESI+	2.308017	↓	
20	Clemastine	Exogenous	382.1341	5.625991667	C06913	ESI+	2.378317	↑	
21	Sphingosine‐1‐phosphate	Endogenous	397.2789	5.02475	C06124	ESI—	1.788306	↑	
22	Sinigrin	Exogenous	398.0009	4.3688	C08427	ESI+	2.293535	↓	
23	Naled	Exogenous	416.7482	4.925633333	C18749	ESI+	2.044597	↓	
24	Lovastatin‐acid	Endogenous	423.2744	4.460641667	C21130	ESI—	2.053382	↓	↓
25	S‐Glutathionyl‐L‐cysteine	Endogenous	427.0941	0.846508333	C05526	ESI—	1.501062	↑	
26	Fortimicin‐A	Exogenous	428.2491	4.002933333	C17708	ESI+	1.400572	↓	
27	Formylmethionyl‐leucyl‐phenylalanine‐methyl‐ester	Exogenous	452.2179	4.146266667	C11221	ESI+	1.905238	↑	
28	DIDS	Exogenous	454.9522	4.440533333	C11591	ESI+	1.765027	↓	
29	3‐Dehydro‐2‐deoxyecdysone	Exogenous	469.293	4.456316667	C16497	ESI—	1.888042	↓	
30	Gentamicin‐C1a	Exogenous	472.2753	4.077933333	C00908	ESI—	2.245466	↓	
31	Loperamide	Exogenous	477.2305	4.073725	C07080	ESI+	2.078937	↓	
32	PD‐123177	Exogenous	481.225	3.9659	C15552	ESI—	1.170123	↑	
33	Terfenadine	Exogenous	489.3459	4.819458333	C07463	ESI+	1.035275	↑	
34	Antibiotic‐JI‐20A	Exogenous	499.3122	4.49535	C17704	ESI+	2.261126	↓	
35	AG‐2034	Exogenous	506.0607	4.591333333	C21280	ESI+	2.496134	↓	
36	dATP	Endogenous	513.9879	4.50535	C00131	ESI+	2.123148	↓	
37	Carpaine	Exogenous	517.3369	5.157225	C10135	ESI+	1.344134	↑	
38	Glycochenodeoxycholate‐7‐sulfate	Endogenous	530.2804	4.149166667	C15559	ESI+	2.230403	↑	
39	Fusidic‐acid	Exogenous	534.3739	4.841966667	C06694	ESI+	2.151472	↑	
40	Terpendole‐C	Exogenous	537.3366	6.36535	C20546	ESI—	2.585171	↓	
41	6,8a‐Seco‐6,8a‐deoxy‐5‐oxoavermectin‐“1b”‐aglycone	Exogenous	555.3294	4.481058333	C11961	ESI—	2.656443	↓	
42	13‐beta‐D‐Glucosyloxydocosanoate	Exogenous	557.3456	4.538316667	C04103	ESI+	2.43886	↓	
43	Ceftiofur‐sodium	Exogenous	567.9966	4.548708333	C13143	ESI+	2.394545	↓	
44	6,8a‐Seco‐6,8a‐deoxy‐5‐oxoavermectin‐“2b”‐aglycone	Exogenous	573.3386	4.546616667	C11953	ESI+	2.426529	↓	
45	Hyperforin	Endogenous	575.3465	4.557583333	C07608	ESI+	2.722633	↓	
46	Cefotetan	Exogenous	576.0131	4.558666667	C06886	ESI+	2.613245	↓	
47	N‐Acetyl‐N6,O‐didemethylpuromycin‐5′‐phosphate	Exogenous	588.1559	8.016608333	C07029	ESI+	3.045418	↑	
48	12‐Ethyl‐8‐isobutyl‐3‐vinylbacteriochlorophyllide‐d	Exogenous	599.2915	5.91175	C21429	ESI—	1.271484	↑	↓
49	PM‐Toxin‐A	Exogenous	602.4637	4.234283333	C08553	ESI+	2.158081	↓	
50	ADP‐5‐ethyl‐4‐methylthiazole‐2‐carboxylate	Exogenous	619.0355	4.5825	C20784	ESI+	2.891763	↓	
51	epsilon‐Rhodomycin‐T	Exogenous	624.1855	8.7453	C18640	ESI+	2.645759	↑	
52	Gambogic acid	Exogenous	629.3144	4.30445	C10062	ESI+	1.796484	↓	
53	Remikiren	Exogenous	631.3556	4.314766667	C07465	ESI+	2.271763	↓	
54	Lopinavir	Exogenous	646.3952	4.587658333	C12871	ESI+	1.646066	↓	
55	Oleanoic‐acid_3‐O‐glucuronide	Exogenous	650.4319	4.736125	C08964	ESI+	1.523996	↓	
56	Amiodarone	Exogenous	663.0626	4.610608333	C06823	ESI+	2.877233	↓	
57	Cefpiramide sodium	Exogenous	673.0667	4.608	C13376	ESI+	1.846436	↓	
58	Guanosine‐3′‐diphosphate‐5′‐triphosphate	Endogenous	683.9296	4.176583333	C04494	ESI+	1.805871	↓	
59	gamma‐L‐Glutamyl‐butirosin‐B	Exogenous	685.3253	4.086083333	C18005	ESI+	1.816766	↑	
60	Rifamycin‐Z	Exogenous	690.2306	7.156833333	C14723	ESI+	2.602993	↑	
61	Rifamycin‐W‐hemiacetal	Exogenous	692.2463	4.461591667	C14722	ESI+	1.600569	↓	
62	Selenodiglutathione	Exogenous	693.074	4.505883333	C18870	ESI+	1.405413	↓	
63	Scillaren‐A	Exogenous	731.3075	4.163466667	C08879	ESI+	2.205564	↑	
64	Rifamycin‐S	Exogenous	734.2554	7.14715	C14540	ESI+	3.616845	↑	
65	Azithromycin	Exogenous	749.5108	6.503883333	C06838	ESI+	1.276195	↑	
66	CoA	Endogenous	768.1264	4.524841667	C00010	ESI+	1.421903	↓	
67	Deltorphin‐C	Endogenous	786.4179	4.371791667	C18097	ESI+	2.16064	↓	
68	Tacrolimus	Exogenous	804.4873	4.5256	C01375	ESI—	2.543604	↓	
69	Gymnemic‐acid‐I	Exogenous	824.4852	4.402683333	C08947	ESI—	2.540749	↓	
70	Erythromycin‐ethylsuccinate	Exogenous	862.5151	4.5558	C12796	ESI+	2.450388	↓	
71	Avermectin‐B2b	Exogenous	877.499	4.490675	C11959	ESI+	2.236289	↓	

Abbreviations: CON (C), control group; MOD (M), model group; m/z, mass‐to‐charge ratio; S: Saorilao‐4 decoction‐treated group; VIP: variables important in projection, VIP values from PLS‐DA model.

**FIGURE 6:**
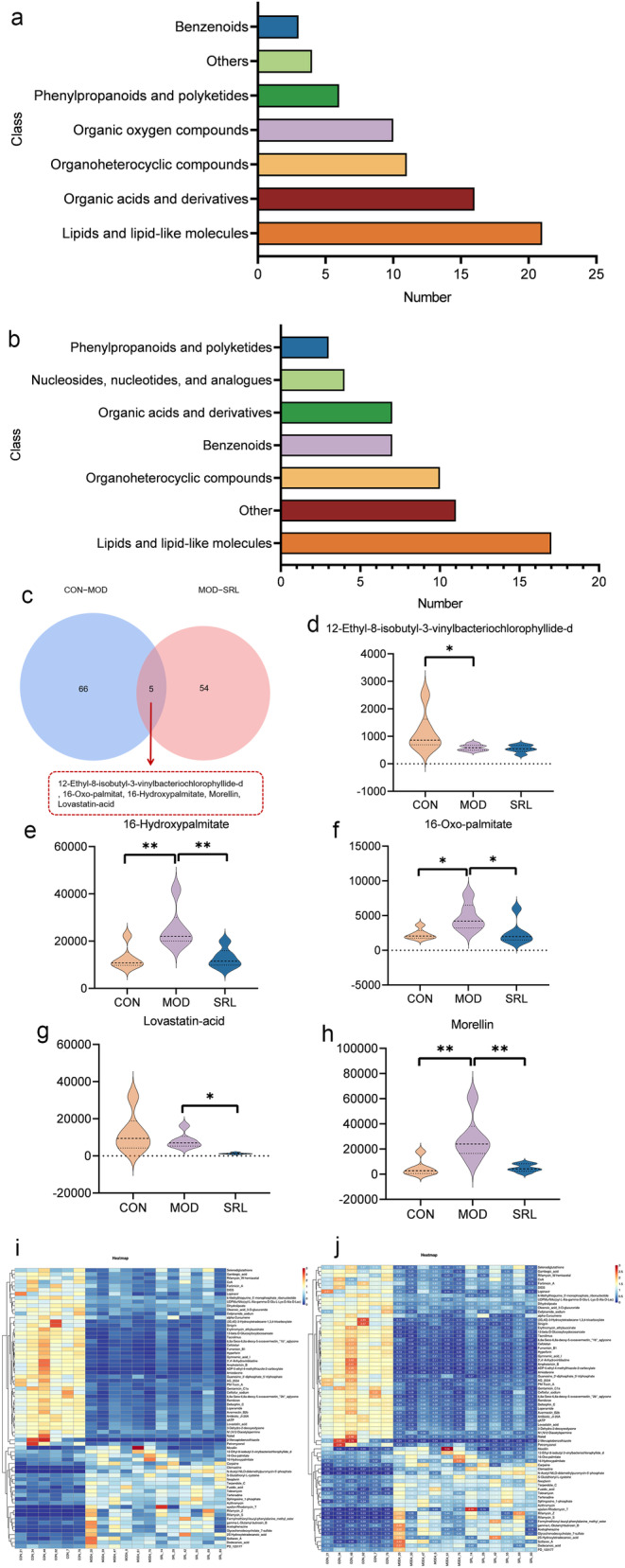
Analysis of differentially expressed metabolites. (a) Classification of 71 potential biomarkers differentially expressed between the CON and MOD groups. (b) Classification of 59 potential biomarkers differentially expressed between the MOD and SRL groups. (c) Venn diagrams of differential metabolites between the CON and MOD groups and the MOD and SRL groups. (d–h) Levels of 16‐oxo‐palmitate, 12‐ethyl‐8‐isobutyl‐3‐vinylbacteriochlorophyllide‐d, 16‐hydroxypalmitate, morellin, and lovastatin‐acid in the experimental groups. (i) Heatmap of the differential abundance of metabolites in each group. Columns represent samples, and rows represent metabolites. Color saturation indicates metabolite expression values, with blue indicating the lowest expression and red the highest. (j) Heatmap of p‐values of serum differential metabolites in each experimental group. Columns represent samples, and rows represent metabolites. Color saturation indicates metabolite expression values, with blue indicating the lowest expression and red the highest. CON, control group; MOD, model group; SRL, SRL‐treated group.

These 71 metabolites can be classified into seven categories: 3 benzene analogs, 21 lipids and related compounds, 16 organic acids and their derivatives, 10 organic oxygen compounds, 11 organic heterocyclic compounds, 6 phenylpropanes and polyketones, and 4 other compounds (Figure 6a). Of the 59 differential metabolites between the MOD and SRL groups, 11 were identified in the positive ion mode and 48 in the negative ion mode (Table 2).

**TABLE 2:** The 59 differential metabolites between the MOD and SRL groups.

No	Metabolites	Source	m/z	Retention time [s]	MS1keggID	Ion mode	VIPa	Regulated (M/S)
1	K‐252c	Exogenous	134.0742804	0.890816667	C21126	ESI—	2.356092404	↓
2	S‐(4‐Methylthiobutylthiohydroximoyl)‐L‐cysteine	Exogenous	167.0326149	2.033891667	C17242	ESI—	2.741823079	↓
3	4‐Sulfobenzyl‐alcohol	Exogenous	187.0069522	2.976533333	C06678	ESI—	3.190218998	↓
4	4‐Chlorophenoxyacetate	Exogenous	202.0263945	3.26255	C07088	ESI—	4.61104982	↑
5	Myristoleic‐acid	Endogenous	225.185699	3.653575	C08322	ESI—	2.160397164	↓
6	Amifostine	Exogenous	230.0731334	2.039216667	C06819	ESI—	4.344288508	↓
7	Neostigmine	Exogenous	239.1656186	4.350266667	C07258	ESI—	3.015238266	↓
8	Pyridoxal‐phosphate	Endogenous	246.0151527	2.700766667	C00018	ESI—	3.03022979	↑
9	Methyl‐farnesoate	Exogenous	249.1855104	5.78365	C16503	ESI—	3.344124393	↓
10	(9Z)‐Hexadecenoic‐acid	Exogenous	253.2172219	3.877825	C08362	ESI—	1.923292478	↓
11	Quetiapine	Exogenous	254.4359555	7.452433333	C07397	ESI—	2.507843656	↓
12	Hexadecanoic acid	Endogenous	255.2331266	7.452275	C00249	ESI—	2.685719155	↓
13	16‐Oxo‐palmitate	Exogenous	269.2129311	7.450716667	C19614	ESI—	2.397946379	↓
14	16‐Hydroxypalmitate	Exogenous	271.2282737	6.427491667	C18218	ESI—	2.236446509	↓
15	Xanthosine	Endogenous	283.0689418	2.29355	C01762	ESI—	2.717312857	↓
16	Phylloquinone	Exogenous	299.2232881	4.76775	C02059	ESI—	1.633094397	↓
17	Emedastine	Exogenous	318.2291174	5.188941667	C07785	ESI—	2.159306484	↓
18	Nebramine	Exogenous	322.2086714	6.761883333	C21259	ESI—	3.703629037	↓
19	13,16‐Docosadienoic‐acid	Endogenous	335.2954169	8.4689	C16533	ESI—	1.904946515	↓
20	Chloramphenicol	Exogenous	338.0308712	1.879716667	C00918	ESI—	2.806764818	↓
21	Mahanimbine	Exogenous	347.2104485	6.416883333	C09220	ESI—	2.055685542	↓
22	Fortimicin‐AP	Exogenous	350.2395162	7.576483333	C17975	ESI—	2.439468163	↓
23	Istamycin‐KL1	Exogenous	352.2196139	5.185383333	C17983	ESI—	2.514847593	↓
24	S‐Adenosylmethioninamine	Endogenous	354.1505804	6.426866667	C01137	ESI—	2.022599521	↓
25	N1‐(5‐Phospho‐alpha‐D‐ribosyl)‐5,6‐dimethylbenzimidazole	Exogenous	357.0831929	2.401308333	C04778	ESI—	2.242775997	↑
26	Fenofibrate	Exogenous	376.1292993	7.449116667	C07586	ESI—	2.408253627	↓
27	Deoxyviolacein	Exogenous	379.0912057	2.801308333	C21133	ESI—	2.847958141	↑
28	Lovastatin‐acid	Endogenous	379.2496536	3.350483333	C21130	ESI—	2.882076811	↓
29	7‐Methylguanosine‐5′‐phosphate	Exogenous	394.1016591	2.0295	C03998	ESI—	3.457369876	↓
30	Mammeisin	Exogenous	405.1740717	7.246125	C09275	ESI—	1.813420217	↓
31	Chlorobiocic‐acid	Exogenous	414.0789011	2.027333333	C12471	ESI—	2.818656358	↓
32	(3R,2′S)‐Myxol‐2′‐(2,4‐di‐O‐methyl‐alpha‐L‐fucoside)	Exogenous	414.2253147	6.993116667	C15937	ESI—	2.339343697	↓
33	Topotecan	Exogenous	420.1557356	5.1869	C11158	ESI—	2.446882303	↓
34	Losartan	Exogenous	421.1538029	5.188683333	C07072	ESI—	2.044814973	↓
35	Bis(glutathionyl)spermine	Endogenous	425.1474935	6.418833333	C16563	ESI—	2.924024988	↓
36	Tylactone	Exogenous	439.2702477	3.36125	C12000	ESI—	2.725359663	↓
37	Nummularine‐F	Exogenous	444.2604102	3.841016667	C10011	ESI—	1.953146617	↓
38	Hydrocortisone‐caproate	Endogenous	459.2730429	3.876383333	C13422	ESI—	2.967504372	↓
39	Lonchocarpenin	Exogenous	464.2111036	6.696966667	C10491	ESI—	1.916531831	↓
40	Vindoline	Endogenous	472.2448691	3.745983333	C01626	ESI—	2.31957194	↓
41	Bacampicillin	Exogenous	481.1794266	6.4188	C08122	ESI—	2.914323802	↓
42	All‐trans‐Retinoyl‐beta‐glucuronide	Endogenous	492.2573429	3.522366667	C11061	ESI—	1.885168132	↓
43	dTDP‐4‐oxo‐2,3,6‐trideoxy‐D‐glucose	Exogenous	513.0705038	2.782041667	C18032	ESI—	2.718022129	↑
44	Terpendole‐K	Exogenous	516.2737314	3.734208333	C20552	ESI—	2.71667853	↓
45	13‐Dihydrocarminomycin	Endogenous	531.2023984	3.880391667	C12431	ESI—	2.223423711	↓
46	Morellin	Exogenous	560.2637513	4.00355	C10085	ESI—	2.741607754	↓
47	27‐O‐Demethylrifamycin‐SV	Exogenous	699.3166999	2.689575	C14727	ESI—	1.720559343	↓
48	Stevioside	Exogenous	820.3949203	2.670125	C09189	ESI—	1.982581016	↓
49	1,2‐Dihydrostilbene	Endogenous	183.1163109	7.610683333	C14685	ESI+	2.802918722	↓
50	(E,E)‐4,8,12‐Trimethyltrideca‐1,3,7,11‐tetraene	Exogenous	219.2105274	8.4746	C20700	ESI+	3.735059766	↓
51	Pentahomomethionine	Exogenous	220.1355083	4.434433333	C17229	ESI+	2.928792223	↓
52	10‐Deoxymethynolide	Exogenous	314.2326957	5.017783333	C11993	ESI+	2.07031908	↓
53	L‐Palmitoylcarnitine	Endogenous	400.3423564	6.529216667	C02990	ESI+	3.510537739	↓
54	3alpha,7alpha,12alpha,26‐Tetrahydroxy‐5beta‐cholestane	Endogenous	454.3878934	7.224383333	C05446	ESI+	3.49533266	↓
55	5alpha‐Cyprinol	Endogenous	470.383035	6.655525	C16890	ESI+	2.878259357	↓
56	Terpendole‐G	Exogenous	490.2322387	3.614016667	C20586	ESI+	1.439185314	↓
57	N‐Acetylleukotriene‐E4	Exogenous	504.2401222	5.442316667	C11361	ESI+	2.089042939	↓
58	Americine	Exogenous	584.2579212	6.253858333	C09996	ESI+	3.005617845	↓
59	12‐Ethyl‐8‐isobutyl‐3‐vinylbacteriochlorophyllide‐d	Exogenous	599.2855108	5.27085	C21429	ESI+	2.15483862	↓

Abbreviations: CON (C), control group; MOD (M), model group; m/z, mass‐to‐charge ratio; S: Saorilao‐4 decoction‐treated group; VIP: variables important in projection, VIP values from PLS‐DA model.

The 59 metabolites can be classified into seven categories: 7 benzene analogs, 17 lipids and lipid‐like molecules, 3 phenylpropanes and polyketides, 4 nucleosides, nucleotides, and analogs, 7 organic acids and their derivatives, 10 organic heterocyclic compounds, and 11 other compounds (Figure 6b). To analyze the distribution of the 71 differential metabolites, Venn diagrams were used. These diagrams compared the components between the CON and MOD groups, as well as the MOD and SRL groups, revealing five overlapping components (Figure 6c). The changes in the levels of these five components were further examined and illustrated using violin plots (Figure 6d–h). The levels of four of these components, including 16‐oxo‐palmitate, 16‐hydroxypalmitate, morellin, and 12‐ethyl‐8‐isobutyl‐3‐vinylbacteriochlorophyllide‐d, were restored in the SRL‐treated group. Heatmaps show the relative increases (red) or decreases (blue) in the levels of differential metabolites when comparing the MOD or SRL groups to the CON group (Figure 6i). *P*‐value heatmaps were employed to visualize the differences among the 71 components between the CON group versus the MOD group and the SRL group versus the MOD group, providing a more intuitive observation of these differences (Figure 6j).

### 3.5 Association Between the Differential Metabolite‐Related Metabolic Pathways and Therapeutic Effects of Saorilao‐4 Decoction‐Treated Group

**FIGURE 7:**
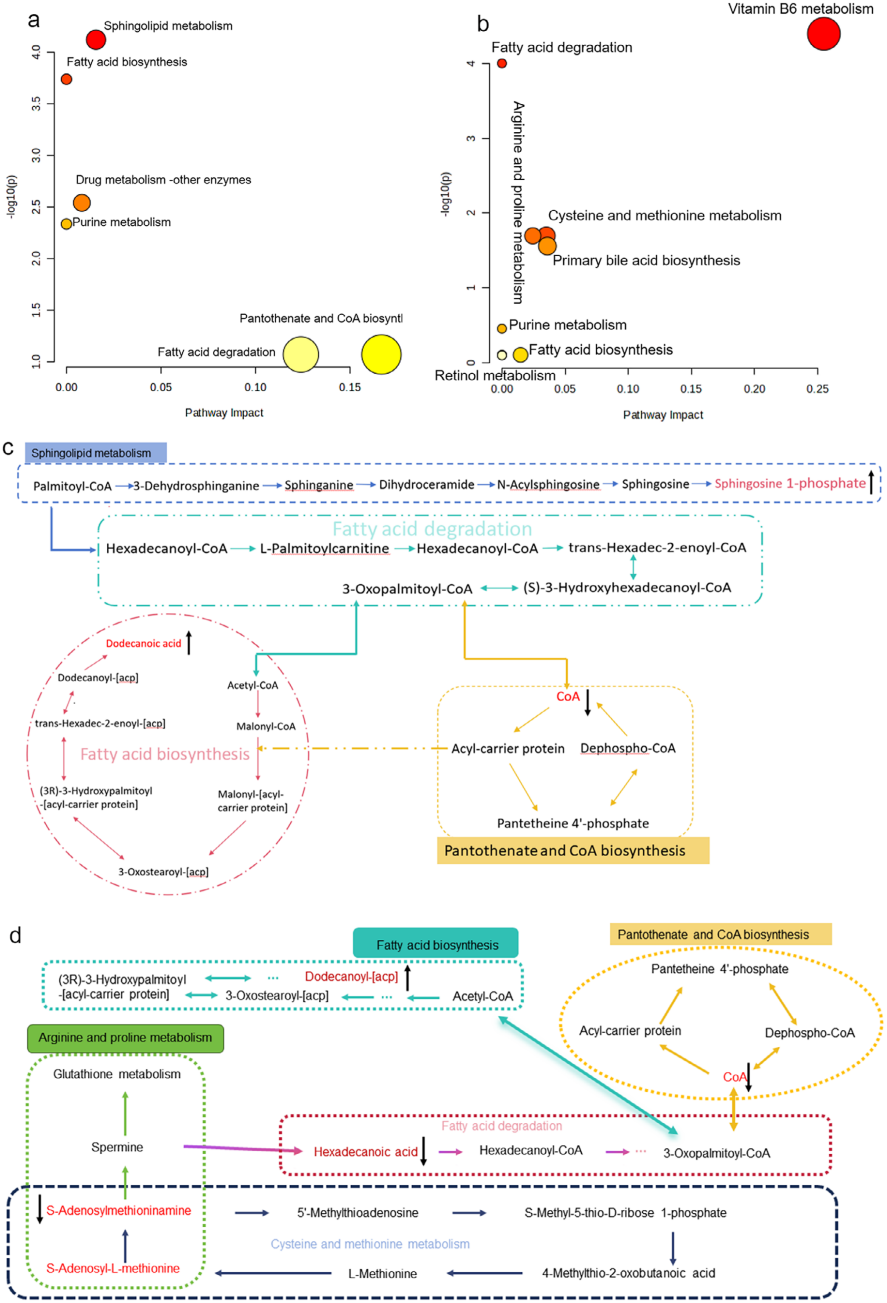
Analysis of potential biomarkers and their associated metabolic pathways. (a) Metabolic pathway alterations identified in the MOD group using metabolomics pathway analysis. The proportions and color of each circle represent the pathway effect value and p‐value, respectively. (b) Network of key biomarkers and pathways impacted by treatment with Saorilao‐4 decoction. (c) Identification of potential pathogenic pathways in PF based on the KEGG pathway database. (d) Identification of potential pathways involved in the therapeutic efficacy of Saorilao‐4 decoction against lung fibrosis. Metabolites in red indicate the main metabolites of each metabolic pathway. ↑ potential biomarkers upgraded; ↓ potential biomarkers downgraded; KEGG, Kyoto Encyclopedia of Genes and Genomes.

We analyzed the correlation between the 71 metabolites using the Pearson rank correlation analysis. We performed functional analysis on the significantly different metabolites between the CON and MOD groups using metabolomics pathway analysis. The findings indicated that these metabolites were primarily associated with sphingosine 1‐phosphate, palmitoyl‐CoA, dodecanoic acid, hexadecanoic acid, and CoA. These metabolites were linked to pathways involved in sphingolipid metabolism, fatty acid biosynthesis, drug metabolism, purine metabolism, pantothenic acid and CoA biosynthesis, and fatty acid degradation, all of which are implicated in the onset of PF (Figure 7a,c).

We conducted KEGG enrichment analysis to assess the impact of SRL on the metabolic pathways of different metabolites between the MOD and SRL groups (Figure 7b,d). The results demonstrated that SRL significantly influenced metabolic pathways such as fatty acid degradation, purine metabolism, cysteine and methionine metabolism, arginine and proline metabolism, and other metabolic pathways in PF model rats. These pathways are closely related to inflammatory factor release, the body's antioxidant capacity, and overall in vivo metabolism. Further analysis indicated that SRL may influence these metabolic processes by downregulating SAM. Additionally, it could mitigate PF‐induced metabolic disorders by reducing fatty acid degradation, as evidenced by a decrease in hexadecanoic acid levels.

## 4 Discussion

PF is a chronic interstitial lung disease characterized by unclear pathogenesis and a lack of precise, targeted treatments (Yan et al. 2023). We used a bleomycin‐induced PF model in rats to study this condition. This model is known for its stability, efficacy, and low mortality. It demonstrates features such as inflammation, collagen deposition, and lung structural damage.

Metabolomics analysis revealed the involvement of several pathways in PF development, including sphingolipid, fatty acid, purine, cysteine, methionine, arginine, and proline metabolism. These pathways and their metabolites could serve as important diagnostic tools for early PF and may also represent potential therapeutic targets.

Among the 71 differentially expressed metabolites identified between the CON and MOD groups, five metabolites were significantly linked to four metabolic pathways. For instance, sphingosine‐1‐phosphate (S1P), generated through sphingosine phosphorylation catalyzed by sphingosine kinase (SK1 or SK2), regulates the alveolar‐capillary endothelial barrier via the S1P1/S1P3 signaling pathway, potentially influencing pulmonary edema (Kennedy et al. 2009). However, S1P has varying effects on alveolar‐capillary permeability depending on its concentration (Qiu et al. 2022). Elevated S1P levels bind to the S1P3 receptor and activate G12/13, stimulating the Rho pathway, leading to stress fiber production and increased vascular permeability (Lee et al. 1999). The lung tissues of rats in the model group showed clear signs of inflammatory cell infiltration and collagen fiber deposition, confirming the successful development of the bleomycin‐induced PF model.

Local activation of IL‐1β plays a central role in mediating the proinflammatory response, leading to the activation of secondary inflammatory mediators. IL‐6 acts systemically on the liver to produce acute‐phase proteins, fibrinogen, and fibrinogen activator inhibitors (Potere et al. 2021), contributing to inflammation and PF. Hyaluronidase modifies the structure and function of the extracellular matrix (ECM) by breaking down hyaluronic acid. Excessive ECM production is a hallmark of fibrotic disease progression, and persistently elevated levels of hyaluronidase indicate uncontrolled fibrosis (Kim and Seki 2023). Serum LN, collagen type III, and collagen type IV, synthesized intracellularly and involved in ECM synthesis, correlate positively with the degree of fibrosis and can be used as fibrosis markers (Liu et al. 2016). Our results indicate that lung IL‐1β, IL‐6, HA, LN, PC‐III, and Col‐IV levels were significantly higher in the MOD group than in the CON group (all *p* < 0.01), while the SRL group exhibited significantly lower levels of these markers than the MOD group (all *p* < 0.01). Collectively, these findings indicate that SRL effectively reduces the inflammatory response and alleviates PF.

The analysis of metabolic components among the CON, MOD, and SRL groups identified five common differential metabolites. Four of these metabolites returned to normal levels after administering SRL. 16‐Oxo‐palmitate, a fatty acid, increased in rats with PF compared to those in the CON group, but its levels returned to nearly normal after treatment with SRL. 16‐Hydroxypalmitate, a hydroxylated fatty acid in the cytochrome P450 family, is involved in fatty acid oxidation. Morellin, an organic pyranoxanthone derivative found in the human intestinal tract, has antimicrobial and antioxidant effects (Wong et al. 2017). Serum levels of morellin were higher in PF rats than in healthy rats, but SRL normalized these levels as PF resolved. 12‐Ethyl‐8‐isobutyl‐3‐vinylbacteriochlorophyllide‐d, a secondary compound in porphyrin metabolism, showed altered levels in PF due to the potential vitamin B6 deficiency and hyperhomocysteinemia (Indika et al. 2023). SRL reduced 12‐ethyl‐8‐isobutyl‐3‐vinylbacteriochlorophyllide‐d levels compared to the MOD group, suggesting the promotion of the vitamin B6 metabolic pathway while inhibiting cysteine and methionine metabolism, contributing to its therapeutic effect in PF.

Abnormal fatty acid metabolism, especially the overproduction of profibrotic lipids such as lysophospholipids and sphingolipids, plays a crucial role in the development of PF (Geng et al. 2022). The three major metabolic pathways—fatty acid biosynthesis, pantothenic acid and CoA biosynthesis, and fatty acid degradation—overlapped between the CON and MOD groups and the SRL and MOD groups, underscoring their significance in the effects of the studied compound. Saturated fatty acid–induced lipotoxicity increases endoplasmic reticulum stress and induces apoptosis (Volmer, van der Ploeg, and Ron 2013). High fatty acid levels may exacerbate PF through increased collagen deposition (Matsuzaka et al. 2007). Our study found that lowering hexadecanoic acid levels inhibited fatty acid and lipid metabolism. This reduction decreased fatty acid accumulation and lowered dodecanoic acid levels, which may help mitigate the potential pathogenesis of PF (Chu et al. 2019).

The metabolism of arginine and proline is crucial for studying PF‐related conditions such as asbestosis, silicosis, fibrosing pulmonary nodular disease, and idiopathic PF (Mirsaeidi et al. 2016). Proline, essential for protein synthesis and metabolism, is essential for wound healing, antioxidant defense, and immune modulation (Wu et al. 2011). Collagen, the main structural protein, heavily depends on proline as its primary constituent. Ornithine, which can be converted to proline and hydroxyproline, is also vital for collagen synthesis (Xue et al. 2021). Ornithine can be decarboxylated by ornithine decarboxylase to produce putrescine, a precursor of arginine. This conversion needs SAM, which supplies essential methyl and adenyl groups (Yiwen et al. 2020). SRL treatment reduces SAM levels compared to the MOD group, thereby inhibiting arginine and proline metabolisms and mitigating PF progression.

As SAM levels drop, fewer methyl groups are available, affecting homocysteine levels. Homocysteine, a sulfur‐containing amino acid, plays a key role in methionine metabolism (Chang et al. 2017). Both of these metabolic pathways are associated with metabolic diseases, particularly oxidative stress (Blachier, Andriamihaja, and Blais 2020). Inhibition of cysteine and methionine metabolism may contribute to the amelioration of PF (Li, Chen, and Zou 2002). Further research on how homocysteine metabolism regulates oxidative stress will improve our understanding of PF pathogenesis and offer new treatment strategies for related diseases.

The literature has reported that sphingolipid metabolic pathways are closely linked to fibrosis in the lungs, kidneys, liver, and heart (Maja et al. 2023; Huwiler and Pfeilschifter 2018). Additionally, fatty acid, purine, cysteine and methionine, and arginine and proline metabolisms are the major pathways involved in PF. In the lungs of PF rats, components such as sphingomyelin 1‐phosphate, hexadecanoic acid, 3‐oxopalmitoyl coenzyme A, and SAM are significantly expressed. These components may serve as potential diagnostic markers. These insights will inform future targeted metabolomics and broader multi‐omics studies.

## 5 Conclusions

In summary, we developed an animal model of bleomycin‐induced PF in rats. Serum metabolomics showed that SRL, a Mongolian medicine, counteracts changes in the blood metabolic profile caused by PF. It demonstrates both therapeutic and preventive effects against this condition. Additionally, SRL mitigates the progression of PF by reducing hexadecanoic acid and SAM levels. These findings clarify the in vivo metabolic mechanisms that underlie the therapeutic effects of SRL in PF. They also identify potential metabolic biomarkers. Future research will involve human samples to validate these metabolic profiles further.

## Author Contributions

**Jiali Liu:** conceptualization (equal), writing – original draft (equal). **Xinni Song:** data curation (equal), writing – original draft (equal). **Xinyue Fu:** data curation (equal). **Shufang Niu:** formal analysis (equal), funding acquisition (equal). **Hong Chang:** methodology (equal). **Songli Shi:** supervision (equal). **Meiqing Yang:** formal analysis (equal), validation (equal). **Peng Wang:** methodology (equal), writing – review and editing (equal). **Wanfu Bai:** funding acquisition (equal), supervision (equal), writing – review and editing (equal).

## Ethics Statement

The animal experiments were approved by the Ethical Committee of Baotou Medical College (no. 2022‐96).

## Conflicts of Interest

The authors declare no conflicts of interest.

## Data Availability

The datasets used and/or analyzed during the current study are available from the corresponding author upon reasonable request.
